# Diabetes mellitus, other medical conditions and familial history of cancer as risk factors for pancreatic cancer

**DOI:** 10.1038/sj.bjc.6690607

**Published:** 1999-08

**Authors:** D T Silverman, M Schiffman, J Everhart, A Goldstein, K D Lillemoe, G M Swanson, A G Schwartz, L M Brown, R S Greenberg, J B Schoenberg, L M Pottern, R N Hoover, J F Fraumeni

**Affiliations:** Division of Cancer Epidemiology and Genetics, National Cancer Institute, Bethesda, MD, USA; Division of Digestive Diseases and Nutrition, National Institute of Diabetes and Digestive and Kidney Diseases, Bethesda, MD, USA; Department of Surgery, The Johns Hopkins School of Medicine, Baltimore, MD, USA; Michigan Cancer Foundation, Detroit, MI, USA; Division of Epidemiology, Emory University School of Public Health, Atlanta, GA, USA; Special Epidemiology Program, New Jersey State Department of Health, Trenton, NJ, USA; Office of the Director, National Institutes of Health, Bethesda, MD, USA

**Keywords:** diabetes mellitus, cholecystectomy, allergies, family history of cancer, pancreatic neoplasm

## Abstract

In a population-based case-control study of pancreatic cancer conducted in three areas of the USA, 484 cases and 2099 controls were interviewed to evaluate the aetiologic role of several medical conditions/interventions, including diabetes mellitus, cholecystectomy, ulcer/gastrectomy and allergic states. We also evaluated risk associated with family history of cancer. Our findings support previous studies indicating that diabetes is a risk factor for pancreatic cancer, as well as a possible complication of the tumour. A significant positive trend in risk with increasing years prior to diagnosis of pancreatic cancer was apparent (*P*-value for test of trend = 0.016), with diabetics diagnosed at least 10 years prior to diagnosis having a significant 50% increased risk. Those treated with insulin had risks similar to those not treated with insulin (odds ratio (OR) = 1.6 and 1.5 respectively), and no trend in risk was associated with increasing duration of insulin treatment. Cholecystectomy also appeared to be a risk factor, as well as a consequence of the malignancy. Subjects with a cholecystectomy at least 20 years prior to the diagnosis of pancreatic cancer experienced a 70% increased risk, which was marginally significant. In contrast, subjects with a history of duodenal or gastric ulcer had little or no elevated risk (OR = 1.2; confidence interval = 0.9–1.6). Those treated by gastrectomy had the same risk as those not receiving surgery, providing little support for the hypothesis that gastrectomy is a risk factor for pancreatic cancer. A significant 40% reduced risk was associated with hay fever, a non-significant 50% decreased risk with allergies to animals, and a non-significant 40% reduced risk with allergies to dust/moulds. These associations, however, may be due to chance since no risk reductions were apparent for asthma or several other types of allergies. In addition, we observed significantly increased risks for subjects reporting a first-degree relative with cancers of the pancreas (OR = 3.2), colon (OR = 1.7) or ovary (OR = 5.3) and non-significantly increased risks for cancers of the endometrium (OR = 1.5) or breast (OR = 1.3). The pattern is consistent with the familial predisposition reported for pancreatic cancer and with the array of tumours associated with hereditary non-polyposis colon cancer. © 1999 Cancer Research Campaign


British Journal of Cancer (1999) 80(11), 1830–1837
© 1999 Cancer Research Campaign
Article no. bjoc.1999.0607

### Diabetes mellitus, other medical conditions and familial history of cancer as risk factors for pancreatic cancer

**DT Silverman[1], M Schiffman[1], J Everhart[2], A Goldstein[1], KD Lillemoe[3], GM Swanson[4,*], AG Schwartz[4,‡], LM Brown[1], RS Greenberg[5,†], JB Schoenberg[6], LM Pottern[7], RN Hoover[1] and JF Fraumeni Jr[1]**

[1]Division of Cancer Epidemiology and Genetics, National Cancer Institute, Bethesda, MD, USA; [2]Division of Digestive Diseases and Nutrition, National Institute of Diabetes and Digestive and Kidney Diseases, Bethesda, MD, USA; [3]Department of Surgery, The Johns Hopkins School of Medicine, Baltimore, MD, USA; [4]Michigan Cancer Foundation, Detroit, MI, USA; [5]Division of Epidemiology, Emory University School of Public Health, Atlanta, GA, USA; [6]Special Epidemiology Program, New Jersey State Department of Health, Trenton, NJ, USA; [7]Office of the Director, National Institutes of Health, Bethesda, MD, USA

**Summary** In a population-based case-control study of pancreatic cancer conducted in three areas of the USA, 484 cases and 2099 controls were interviewed to evaluate the aetiologic role of several medical conditions/interventions, including diabetes mellitus, cholecystectomy, ulcer/gastrectomy and allergic states. We also evaluated risk associated with family history of cancer. Our findings support previous studies indicating that diabetes is a risk factor for pancreatic cancer, as well as a possible complication of the tumour. A significant positive trend in risk with increasing years prior to diagnosis of pancreatic cancer was apparent (*P*-value for test of trend = 0.016), with diabetics diagnosed at least 10 years prior to diagnosis having a significant 50% increased risk. Those treated with insulin had risks similar to those not treated with insulin (odds ratio (OR) = 1.6 and 1.5 respectively), and no trend in risk was associated with increasing duration of insulin treatment. Cholecystectomy also appeared to be a risk factor, as well as a consequence of the malignancy. Subjects with a cholecystectomy at least 20 years prior to the diagnosis of pancreatic cancer experienced a 70% increased risk, which was marginally significant. In contrast, subjects with a history of duodenal or gastric ulcer had little or no elevated risk (OR = 1.2; confidence interval = 0.9–1.6). Those treated by gastrectomy had the same risk as those not receiving surgery, providing little support for the hypothesis that gastrectomy is a risk factor for pancreatic cancer. A significant 40% reduced risk was associated with hay fever, a non-significant 50% decreased risk with allergies to animals, and a non-significant 40% reduced risk with allergies to dust/moulds. These associations, however, may be due to chance since no risk reductions were apparent for asthma or several other types of allergies. In addition, we observed significantly increased risks for subjects reporting a first-degree relative with cancers of the pancreas (OR = 3.2), colon (OR = 1.7) or ovary (OR = 5.3) and non-significantly increased risks for cancers of the endometrium (OR = 1.5) or breast (OR = 1.3). The pattern is consistent with the familial predisposition reported for pancreatic cancer and with the array of tumours associated with hereditary non-polyposis colon cancer.

**Keywords:** diabetes mellitus; cholecystectomy; allergies; family history of cancer; pancreatic neoplasm

Pancreatic cancer, the fifth leading cause of death from cancer in the USA, is expected to cause nearly 30 000 deaths in 1998 (Landis et al, 1998). Despite the high mortality attributable to pancreatic cancer, its aetiology is poorly understood. Cigarette smoking is the only generally accepted risk factor, but explains only about 25% of the disease (Silverman et al, 1994). Several medical conditions have been reported to be associated with risk, but results have been inconsistent across studies. Most persuasive is the risk associated with diabetes mellitus (Everhart and Wright, 1995), with more limited evidence suggesting excess risk for gall-bladder disease or cholecystectomy, chronic calcifying pancreatitis and ulcer or gastrectomy (Anderson et al, 1996). A history of pancreatic cancer among first-degree relatives also has been associated with elevated risk, while protective effects have been reported for asthma and other allergies (Anderson et al, 1996). The lack of consistency of results pertaining to pre-existing medical conditions may be due to misclassification of information because of the predominance of next-of-kin interviews in case-control studies of pancreatic cancer. The rapidly fatal course of this cancer has made it difficult to conduct case-control studies based exclusively on direct interviews with the subjects in a population-based setting.

Our purpose was to conduct a large population-based case-control study based on direct interviews and to evaluate medical conditions/interventions and family history of cancer as risk factors for pancreatic cancer.

*Received 11 August 1998*
*Revised 14 January 1999*
*Accepted 18 January 1999*

*Correspondence to:* DT Silverman, Division of Cancer Epidemiology and Genetics, National Cancer Institute, Executive Plaza South, Room 8108, Bethesda, MD, USA 20892-7240

### MATERIALS AND METHODS

We conducted a population-based case-control study of malignancies that occur excessively in blacks (i.e. cancers of the pancreas, prostate, oesophagus and multiple myeloma) in three areas of the USA. One general population control group was the source of controls for all four types of cancer.

The case series in this analysis included all cases of carcinoma of the pancreas (International Classification of Diseases for Oncology code = 157) first diagnosed from August 1986 to April 1989 among 30- to 79-year-old residents of geographic areas

Present addresses: *College of Human Medicine, Michigan State University, Lansing, MI, USA; †Medical University of South Carolina, Charleston, SC, USA; ‡MCP-Hahnemann School of Medicine, Allegheny University of the Health Sciences, Pittsburgh, PA, USA.

covered by population-based cancer registries located in Atlanta (DeKalb and Fulton counties), Detroit (Macomb, Oakland and Wayne counties) and New Jersey (ten counties). To ensure both the population-based nature of the case series and the completeness of case ascertainment, all cases with reported pancreatic cancer, regardless of the presence of tissue confirmation, were initially included. Because about 15% of the cases lacked tissue confirmation, an in-depth medical chart review was conducted to determine the accuracy of diagnosis. Based on this review, 5.5% of identified pancreatic cancer patients were excluded because they were found to be 'unlikely' to have pancreatic cancer. Additional details regarding the chart review are reported in a previous publication (Silverman et al, 1996).

Because of the rapidly fatal course of pancreatic cancer, death was the main reason for non-participation. Despite our emphasis on identifying and interviewing patients as quickly as possible (median time from diagnosis to interview: 7 weeks), 471 of the 1153 patients initially identified for study died before the interview could be conducted. Of the 682 living cases identified for study, 526 (77%) were interviewed.

To evaluate the comparability of those who died to those who lived long enough to be interviewed, we interviewed next of kin of a sample of 325 deceased cases. The next-of-kin interview included only broad categorical questions that next-of-kin respondents have been shown to answer reliably (McLaughlin et al, 1990; Gavalda et al, 1995). For most questions, the pattern of responses from next of kin of deceased cases was similar to that for personally interviewed cases, including responses to questions on 'ever smoked cigarettes', 'ever drank coffee regularly', 'ever drank alcohol regularly' and 'ever having been told by a physician that they had a specified medical condition/intervention'. For medical conditions/interventions, the percentages of next of kin and directly interviewed respondents who reported the case as having the following condition/intervention were: diabetes mellitus (19% and 18% respectively), cholecystectomy (17% and 11% respectively), ulcer (14% and 16% respectively), asthma (9% and 6% respectively), hay fever (19% and 12% respectively) and eczema (7% and 5% respectively). These data suggest that interviewed cases were probably representative of the total case series with respect to most medical conditions/interventions. For cholecystectomy, asthma and hay fever, however, next of kin reported higher percentages than directly interviewed subjects. Possible under-reporting of such conditions/interventions by interviewed cases may have resulted in underestimation of the risks for these conditions/interventions.

The control series was drawn from the general population of the study areas, frequency matching controls to the expected age-race-gender distribution of cases of all four types of cancer combined in each study area. Controls 30–64 years old were selected by random-digit dialing (Waksberg, 1978). Of the 17 746 households telephoned, 86% provided a household census that served as the sampling frame for selection of controls under age 65 years. Of the 1568 controls chosen from these households, we interviewed 1227 (78%). Controls aged 65–79 years consisted of a stratified random sample drawn from the Health Care Financing Administration rosters of the population age 65 or older in each study area. Of the 1232 older controls selected, we interviewed 926 (75%).

Subjects were usually interviewed at home by interviewers who were not informed of either the case or control status of the subject or the hypotheses under study. Prior to interview, written informed consent to participate in the study was obtained from each subject.

**Table 1** Number of cases and controls and odds ratios for pancreatic cancer according to history of diabetes mellitus, cholecystectomy and ulcer

| Condition/Intervention | No of cases | No of controls | OR[a] | 95% CI |
|---|---|---|---|---|
| Diabetes mellitus[b] | | | | |
| Interval between onset and diagnosis of cancer (years) | | | | |
| No diabetes | 398 | 1846 | 1.0 | |
| 0–1 | 4 | 13 | 1.3 | 0.4–4.0 |
| 2–4 | 18 | 56 | 1.4 | 0.7–2.4 |
| 5–9 | 22 | 54 | 1.7 | 1.0–2.9 |
| ≥10 | 42 | 127 | 1.5 | 1.01–2.2 |
| Ever treated with insulin[c] | | | | |
| No diabetes | 398 | 1846 | 1.0 | |
| No | 31 | 96 | 1.5 | 1.0–2.3 |
| Yes | 33 | 85 | 1.6 | 1.04–2.5 |
| Duration of insulin treatment (years) | | | | |
| No diabetes | 398 | 1846 | 1.0 | |
| 0–4 | 10 | 19 | 2.6 | 1.1–5.8 |
| 5–9 | 11 | 18 | 2.3 | 1.1–5.1 |
| 10–19 | 5 | 35 | 0.7 | 0.3–1.8 |
| ≥20 | 3 | 12 | 0.9 | 0.2–3.3 |
| Cholecystectomy | | | | |
| Interval between surgery and diagnosis of cancer (years) | | | | |
| No cholecystectomy | 345 | 1941 | 1.0 | |
| 0–1 | 78 | 8 | 57.9 | 27.3–123.0 |
| 2–4 | 8 | 15 | 2.7 | 1.1–6.6 |
| 5–9 | 11 | 30 | 1.9 | 0.9–3.9 |
| 10–19 | 14 | 50 | 1.4 | 0.7–2.6 |
| ≥20 | 21 | 47 | 1.7 | 1.0–3.0 |
| Ulcer | | | | |
| History of ulcer[d] | | | | |
| No | 407 | 1821 | 1.0 | |
| Yes | 76 | 277 | 1.2 | 0.9–1.6 |
| Ulcer treated by gastrectomy[d] | | | | |
| No ulcer | 407 | 1821 | 1.0 | |
| Ulcer without gastrectomy | 60 | 233 | 1.2 | 0.9–1.6 |
| Ulcer with gastrectomy | 10 | 39 | 1.2 | 0.6–2.5 |

[a]Odds ratios adjusted for age at diagnosis/interview, race, gender, area, cigarette smoking, alcohol consumption, body mass index and calories from food. [b]Excludes three controls diagnosed with diabetes mellitus before age 20 years. [c]Excludes subjects diagnosed with diabetes mellitus less than 5 years prior to the diagnosis of pancreatic cancer. [d]Odds ratios also adjusted for income (men) and marital status (women).

The questionnaire was designed to elicit detailed information on medical conditions/interventions, family history of cancer, smoking habits, alcohol consumption, coffee and tea drinking, nutritional/dietary factors, usual occupation, and socioeconomic status. Subjects were also queried about their usual adult height and weight, which was used to compute body mass index (BMI) (kg $m^{-2}$ for men, kg $m^{-1.5}$ for women) (Micozzi et al, 1986). The effects of nutritional/dietary factors, cigarette smoking and alcohol drinking on pancreatic cancer risk in this study have been reported in earlier publications (Silverman et al, 1994, 1995, 1998).

We queried subjects to obtain detailed information on the following medical conditions and interventions: diabetes mellitus, cholecystectomy, pancreatitis, ulcer, gastrectomy and allergies. We obtained information on cholecystectomy, rather than cholelithiasis, because we believed recall of cholecystectomy would be more accurate than that of cholelithiasis. Subjects were asked questions regarding their age at onset and duration of each medical condition and their age at the time of each surgery. All

*© 1999 Cancer Research Campaign* 

**Table 2** Odds ratios for pancreatic cancer according to history of diabetes mellitus and body mass index

| | Body Mass Index[a,b] | | | |
|---|---|---|---|---|
| **History of diabetes** | **1** | **2** | **3** | **4** |
| No | | | | |
| OR | 1.0[c] | 1.2 | 1.4 | 1.6 |
| 95% CI | – | 0.8–1.6 | 1.1–2.0 | 1.2–2.2 |
| No. cases/No. controls | 90/503 | 100/499 | 112/462 | 118/451 |
| Yes[d] | | | | |
| OR | 2.7 | 1.7 | 1.8 | 2.2 |
| 95% CI | 1.3–6.0 | 0.7–3.9 | 1.0–3.4 | 1.3–3.7 |
| No. cases/No. controls | 11/22 | 8/28 | 16/55 | 29/76 |

[a]BMI = weight height$^2$ for men; BMI = weight height$^{1.5}$ for women. [b]Quartile cut-points for BMI (based on controls) – men: 17.4–23.1; 23.2–25.1; 25.2–27.2; >27.2 (kg/m$^{-2}$); women: 20.5–27.5; 27.6–30.2; 30.3–34.2; ≥34.4 (kg/m$^{-1.5}$). [c]Odds ratios adjusted for age at diagnosis/interview, race, gender, area, cigarette smoking, alcohol consumption and calories from food. [d]Excludes subjects diagnosed with diabetes less than 5 years prior to diagnosis of pancreatic cancer.

**Table 3** Odds ratios for pancreatic cancer according to history of cholecystectomy and body mass index

| | Body Mass Index[a,b] | | | |
|---|---|---|---|---|
| **History of cholecystectomy** | **1** | **2** | **3** | **4** |
| No | | | | |
| OR | 1.0[c] | 1.0 | 1.1 | 1.3 |
| 95% CI | – | 0.7–1.4 | 0.8–1.6 | 0.9–1.8 |
| No cases/No. controls | 84/494 | 83/497 | 83/476 | 95/474 |
| Yes[d] | | | | |
| OR | 0.8 | 1.9 | 1.7 | 2.6 |
| 95% CI | 0.3–2.4 | 0.8–4.5 | 0.8–3.5 | 1.5–4.6 |
| No cases/No. controls | 4/26 | 8/22 | 12/34 | 22/45 |

[a] BMI = weight height$^2$ for men; BMI = weight height$^{1.5}$ for women. [b]Quartile cut-points for BMI (based on controls) – men: 17.4–23.1; 23.2–25.1; 25.2–27.2; >27.2 (kg/m$^{-2}$); women: 20.5–27.5; 27.6–30.2; 30.3–34.2; ≥34.4 (kg/m$^{-1.5}$). [c]Odds ratios adjusted for age at diagnosis/interview, race, gender, area, cigarette smoking, alcohol consumption and calories from food. [d]Excludes subjects who received a cholecystectomy less then 5 years prior to the diagnosis of pancreatic cancer.

analyses pertaining to diabetes mellitus were restricted to subjects with adult onset diabetes because only three subjects (zero cases, three controls) reported onset of diabetes before age 20. We also obtained detailed information on the use of insulin and other drugs to treat diabetes mellitus. With regard to allergies, we elicited detailed information on all allergies, including hay fever, asthma, eczema, allergies to insect bites, food, animals, drugs, dusts/mould, household products and cosmetics. Medications used to treat asthma, including the use of bronchodilators and steroids, also were obtained. Only seven cases and ten controls reported a hospitalization for pancreatitis, prohibiting any detailed analysis of this potential risk factor.

With regard to family history of cancer, subjects were asked whether any first-degree blood relative ever had cancer, and if so, to specify the type of neoplasm. We did not, however, enumerate all members of the immediate family. Risk associated with family history of cancer in the children of subjects was not included because the number of subjects reporting children with cancer was small.

The effects of medical conditions/interventions and family history of cancer on pancreatic cancer risk were quantified by the odds ratio (OR). ORs and 95% confidence intervals (CIs) were estimated by unconditional logistic regression analysis (Breslow and Day, 1980; Dixon et al, 1990). Statistical models included terms for exposure (i.e. diabetes mellitus, cholecystectomy, ulcer, gastrectomy, or allergies), the matching factors (i.e. age at diagnosis/interview, race, gender and study area), as well as terms for potential confounding variable (i.e. cigarette smoking, alcohol consumption, body mass index, caloric intake, income (men) and marital status (women)). For family history of cancer, only the matching factors were included in the models because additional adjustment for smoking, alcohol consumption and other potential confounding factors had little or no impact on estimates of risk. To test for trend, the exposure variable was treated as continuous by entering the median value for each level of the categorical variable among the controls.

Interviewed subjects were excluded from analysis for the following reasons: unlikely diagnosis of pancreatic cancer (16 cases), presence of islet cell carcinoma (ten cases), no medical record available for review (six cases), unsatisfactory interview (one case and seven controls), and missing data (nine cases and 47 controls). Thus, the analysis of medical conditions/interventions was based on first-person interviews with 484 ‘likely’ cases with a diagnosis of carcinoma of the exocrine pancreas and 2099 population controls.

### RESULTS

### Diabetes mellitus

Table 1 shows risk of pancreatic cancer by length of the interval between diagnosis of diabetes and cancer. A significant positive trend in risk with increasing years prior to diagnosis of cancer was apparent ($P$ = 0.016). Risk was slightly elevated for subjects with onset of diabetes within 1 year of diagnosis of cancer (OR = 1.3; CI = 0.4–4.0). For subjects with longer intervals, risks were significantly increased, with ORs of 1.7 (CI = 1.01–2.9) and 1.5 (CI = 1.01–2.2) for those with onset of diabetes within 5–9 years and 10 or more years before the diagnosis of cancer respectively.

Treatment for diabetes was unrelated to pancreatic cancer risk. Compared to non-diabetics, diabetics who were treated with insulin had an OR of 1.6 (CI = 1.04–2.5), while those who were not treated with insulin had an OR of 1.5 (CI = 1.0–2.3). There was no trend in risk with increased duration of insulin use (Table 1). Diabetics treated with oral medications (OR = 1.4; CI = 0.8–2.6) had risks similar to those who did not (OR = 1.5, CI = 1.1–2.2), while those with special dietary restrictions had a non-significantly higher risk than those without such restrictions (OR = 1.7 and 1.4 respectively). There was only one case and six controls with diabetes who did not have any form of treatment.

Family history of diabetes did not appear to be related to risk of pancreatic cancer. Compared to subjects with no family history, diabetics with a positive family history had an OR of 0.8, while non-diabetics with a positive family history had an OR of 1.0.

Because obesity, an important risk factor for diabetes, has been linked to pancreatic cancer risk in our study (Silverman et al, 1998) as well as others (Friedman and van den Eeden, 1993; Moller et al, 1994; Shibata et al, 1994; Ji et al, 1996), we cross-

 *© 1999 Cancer Research Campaign*

**Table 4** Number of cases and controls and odds ratios for pancreatic cancer according to history of hay fever, asthma and other allergic conditions

| Allergic condition | No. of cases | No. of controls | OR[a] | 95% CI |
|---|---|---|---|---|
| No allergic condition | 277 | 1180 | 1.0 | |
| Any allergic condition | 76 | 488 | 0.7 | 0.5–0.9 |
| Hay fever | 58 | 403 | 0.6 | 0.5–0.9 |
| Asthma | 29 | 117 | 1.0 | 0.6–1.5 |
| Eczema | 23 | 78 | 1.1 | 0.7–1.9 |
| Animal allergy | 6 | 54 | 0.5 | 0.2–1.1 |
| Insect bite/sting allergy | 37 | 181 | 0.8 | 0.6–1.2 |
| Dust or mold allergy | 13 | 98 | 0.6 | 0.3–1.1 |
| Drug allergy | 81 | 215 | 1.4 | 1.0–1.9 |
| Household products allergy | 15 | 36 | 1.5 | 0.8–2.9 |

[a]Odds ratios adjusted for age at diagnosis/interview, race, gender, area, cigarette smoking, alcohol consumption, body mass index and calories from food. CI, confidence intervals.

**Table 5** Number of cases and controls and odds ratios for pancreatic cancer according to treatment for allergic conditions

| Treatment | No. of cases | No. of controls | OR[a] | 95% CI |
|---|---|---|---|---|
| Allergy shots | | | | |
| No allergic conditions | 277 | 1180 | 1.0 | |
| Never allergy shots | 148 | 703 | 0.9 | 0.7–1.1 |
| Ever allergy shots | 23 | 124 | 0.8 | 0.5–1.3 |
| Number of allergy shots | | | | |
| 0 | 425 | 1883 | 1.0 | |
| <10 | 8 | 37 | 1.0 | 0.5–2.2 |
| 10–49 | 6 | 35 | 0.9 | 0.4–2.2 |
| 50–99 | 1 | 13 | 0.4 | 0.1–2.8 |
| ≥100 | 8 | 39 | 0.9 | 0.4–2.0 |
| Asthma treatment | | | | |
| No bronchodilators or steroids | 11 | 41 | 1.0 | |
| Bronchodilators, no steroids | 7 | 43 | 0.7 | 0.2–2.3 |
| Steroids, no bronchodilators | 4 | 8 | 2.5 | 0.5–13.9 |
| Bronchodilators and steroids | 5 | 14 | 1.1 | 0.2–4.5 |

[a]Odds ratios adjusted for age at diagnosis/interview, race, gender, cigarette smoking, alcohol consumption, body mass index and calories from food. CI, confidence intervals.

**Table 6** Number of cases and controls and odds ratios for pancreatic cancer according to family history of cancer[a]

| Site of cancer in first-degree relatives | No. of cases | No. of controls | OR[b] | 95% CI |
|---|---|---|---|---|
| All sites combined | 218 | 784 | 1.3 | 1.1–1.6 |
| Pancreas | 23 | 31 | 3.2 | 1.8–5.6 |
| Oesophagus | 2 | 14 | 0.6 | 0.1–2.9 |
| Stomach | 27 | 120 | 0.9 | 0.6–1.4 |
| Colon | 36 | 91 | 1.7 | 1.1–2.5 |
| Liver | 12 | 37 | 1.4 | 0.7–2.7 |
| Lung | 30 | 111 | 1.1 | 0.7–1.7 |
| Breast | 40 | 128 | 1.3 | 0.9–1.9 |
| Ovary | 5 | 4 | 5.3 | 1.4–20.2 |
| Endometrium | 15 | 41 | 1.5 | 0.8–2.8 |
| Prostate | 11 | 49 | 1.0 | 0.5–1.9 |

[a]Excludes 25 cases and 39 controls with missing information on family history of cancer. [b]Odds ratios relative to a risk of 1.0 for subjects with no family history of cancer, adjusted for age at diagnosis/interview, race, gender and area. CI, confidence intervals.

classified risk simultaneously by history of diabetes and BMI in Table 2. Within each level of BMI, diabetics had a higher risk than non-diabetics. In addition, a significant positive trend in risk with increasing BMI was apparent for non-diabetics ($P$ = 0.02), but not for diabetics. These trends, however, were not significantly different from each other ($P$ > 0.05).

### Cholecystectomy

Table 1 presents risk following cholecystectomy according to the length of the interval to diagnosis of pancreatic cancer. Within 1 year prior to the cancer diagnosis, the risk associated with cholecystectomy was extremely high (OR = 57.9, CI 27.3–123.0). Although much diminished, risk remained elevated with increasing years prior to the diagnosis of cancer. Subjects who had a cholecystectomy 20 or more years prior to the cancer diagnosis had an OR of 1.7 (CI = 1.0–3.0).

Because obesity is a risk factor for cholelithiasis, we cross-classified pancreatic cancer risk by history of cholecystectomy and BMI (Table 3). Within each level of BMI except the first quartile, subjects with cholecystectomy experienced an excess risk compared to those who did not have this procedure. Cholecystectomy also appeared to modify the BMI effect. The risk gradient associated with BMI was stronger among subjects with cholecystectomy than among those without, but these gradients were not significantly different from each other ($P$ > 0.05). Risk for subjects in the top BMI quartile was higher for those with a cholecystectomy (OR = 2.6, CI = 1.5–4.6) than for those without (OR = 1.3, CI = 0.9–1.8).

### Ulcer/gastrectomy

Little or no excess risk was associated with having had a duodenal or gastric ulcer (OR = 1.2, CI = 0.9–1.6) (Table 1). Subjects treated by full or partial gastrectomy had the same risk (OR = 1.2) as those without gastrectomy (OR = 1.2).

### Allergies

Table 4 shows risk of pancreatic cancer by history of allergies. Subjects with a history of any allergic condition had a significantly reduced risk (OR = 0.7, CI = 0.5–0.9). This overall risk reduction, however, was mainly due to a decreased risk among subjects with a history of hay fever, although non-significant decreased risks also were seen among subjects with allergies to animals or to dust or moulds. No protective effects were associated with a history of asthma, eczema or allergy to insect bites/stings. Increased risks were observed for subjects with allergies to drugs (OR = 1.6, CI = 1.2–2.1) and for those with allergies to household products (OR = 1.7, CI = 0.9–3.1).

Compared to subjects without allergies, those who had allergy shots had risks similar to those who never had allergy shots, with no consistent trend in risk with increasing number of shots (Table 5). No significantly decreased risks were observed among asthmatics who used bronchodilators or steroids or both compared to subjects without allergies.

### Family history of cancer

Table 6 gives risk of pancreatic cancer by history of cancer among first-degree relatives. A significant 30% increased risk was associated with a family history of any cancer. Subjects with a family history of pancreatic cancer had a significantly elevated risk (OR = 3.2, CI = 1.8–5.6). This risk was higher for those with pancreatic cancer in a sibling (OR = 3.6, CI = 1.5–8.7) than in a parent (OR = 2.6, CI = 1.2–5.4). Family history of pancreatic cancer was associated with a higher risk among long-term smokers (i.e. smokers for 20 or more years) (OR = 5.3, CI = 2.1–13.4) than non-smokers/short- and moderate-term smokers (i.e. smoked for less than 20 years) (OR = 2.2, CI = 1.0–7.9), although the interaction between familial predisposition and smoking was not statistically significant ($P > 0.05$). Risk associated with familial occurrence did not vary by age at diagnosis/interview of the subject. In fact, point estimates for subjects aged 45–64 years and those aged 65 years or older were identical (OR = 3.3), with CIs of 1.4–7.8 and 1.5–7.2 respectively. There were no cases and two controls under age 45 at diagnosis/interview who reported pancreatic cancer in a first-degree relative.

With regard to family history of other cancers (Table 6), risk of pancreatic cancer was significantly elevated for subjects with a family history of cancers of the colon (OR = 1.7) or ovary (OR = 5.3), and non-significantly elevated for those with a family history of cancers of the endometrium (OR = 1.5), breast (OR = 1.3), or liver (OR = 1.4).

### DISCUSSION

Our findings indicate that diabetes mellitus is a risk factor for pancreatic cancer, as well as a potential consequence of pancreatic cancer. In particular, subjects diagnosed with diabetes at least 10 years prior to the diagnosis of cancer had a significant 50% increased risk, while a non-significant 30% increased risk was seen for subjects whose diabetes was detected within 1 year of the diagnosis of cancer.

The association between diabetes mellitus and pancreatic cancer has been evaluated in more than 30 studies (Green and Jensen, 1985; Chow et al, 1995; Everhart and Wright, 1995; Wideroff et al, 1997; Calle et al, 1998), with most indicating a positive relationship. The critical question has been whether diabetes is a true aetiologic factor or a consequence of pancreatic cancer during a prediagnostic stage. When diabetes arises around the time of cancer diagnosis, it is usually characterized by marked insulin resistance with hyperinsulinaemia that declines after tumour resection (Permert et al, 1994). In 1994, Permert and colleagues showed that, among pancreatic cancer patients, those with diabetes have elevated plasma levels of islet amyloid polypeptide (IAPP), a hormonal factor secreted by B-cells, which may cause insulin resistance and contribute to diabetes in these patients (Permert et al, 1994).

Our study and others indicate that diabetes is also a risk factor for pancreatic cancer. In 1995, Everhart and Wright reported results of a meta-analysis of pancreatic cancer studies and found that diabetics diagnosed at least 5 years prior to the diagnosis of cancer had a relative risk (RR) of 2.0 (CI = 1.2–3.2). The pooled estimate from the cohort studies (RR = 2.6) was higher than that from the case-control studies (OR = 1.8). Our results are consistent with the pooled estimate for case-control studies.

The mechanism by which long-standing diabetes causes pancreatic cancer is uncertain. One possibility is that the relation is mediated by obesity, which is a risk factor for diabetes (Colditz et al, 1995) and pancreatic cancer in the present study (Silverman et al, 1998) as well as previous studies (Friedman and van den Eeden, 1993; Moller et al, 1994; Shibata et al, 1994; Ji et al, 1996). We found, however, that diabetes was related to risk of pancreatic cancer within each quartile of BMI, most notably in the lowest BMI quartile (170% increased risk).

Another possible mechanism derives from experimental studies indicating that exposure to insulin promotes growth in human pancreatic cell lines (Fisher et al, 1996). Hyperinsulinaemia is characteristic of both obesity and non-insulin-dependent diabetes mellitus (NIDDM) (DeFronzo et al, 1997), and may play a key role in pancreatic carcinogenesis (Everhart and Wright, 1995). Among non-diabetics, resistance to insulin action typically increases with increasing BMI, which results in hyperinsulinaemia. Among diabetics, insulin levels are not strongly correlated with BMI, but depend mainly on the degree of impaired B-cell function and hyperglycaemia. The role of hyperinsulinaemia in pancreatic cancer risk is consistent with our finding that the diabetes-related risk was not influenced by obesity, while the risk associated with increasing BMI was seen only in non-diabetics. To further test this hypothesis will require a large-scale cohort study with repeated serum collections to determine if and when insulin levels become elevated prior to the onset of pancreatic cancer.

A third possible mechanism is based on experimental observations that peripheral insulin resistance is associated with increased cell turnover of the pancreatic islets, and stimulation of islet cell proliferation enhances pancreatic carcinogenesis in Syrian hamsters (Pour and Kazakoff, 1996), which is considered an excellent animal model for human pancreatic carcinogenesis (Kazakoff et al, 1996).

Lastly, the observed association between long-standing diabetes and pancreatic cancer may be due to confounding by a correlated variable. This explanation seems unlikely because the OR for diabetes was adjusted for age at diagnosis/interview, race, gender, geographic area, cigarette smoking, alcohol consumption, BMI and calories from food. Adjustment for other potential confounders had little or no impact. Moreover, the relation between long-standing diabetes and pancreatic cancer has been found in at least 20 previous studies conducted in a variety of populations over time (Chow et al, 1995; Everhart and Wright, 1995; Wideroff et al, 1997), diminishing the likelihood that the observed effect is due to confounding.

Another key finding of our study was the positive association between cholecystectomy and pancreatic cancer risk. The 57-fold increased risk for subjects with cholecystectomy within 1 year of cancer diagnosis is probably an artifact resulting from either surgery prompted by symptoms of pancreatic cancer or inaccurate recall of patients regarding diagnostic work-up or treatment. Risk, however, remained elevated with increasing years prior to the diagnosis of malignancy, with a 70% excess risk among subjects with cholecystectomy 20 or more years prior to diagnosis of pancreatic cancer. According to our next-of-kin results (see Materials and Methods), the true magnitude of this risk may, in fact, have been underestimated. Our results suggest that cholecystectomy is a risk factor for pancreatic cancer, as well as a consequence of the malignancy.

These findings are consistent with most studies of cholecystectomy/gallbladder disease and pancreatic cancer. Of at least 17 studies conducted to date, 11 have been positive (Wynder et al, 1973; La Vecchia et al, 1980; Lin and Kessler, 1981; Norell and Ahlbom, 1986; Hyvarinen and Partanen, 1987; Cuzick and Babiker, 1989; Kalapothaki et al, 1993; Shibata et al, 1994; Ekbom et al, 1996; Johanson et al, 1996; Chow et al, 1998). Few studies, however, have considered timing of the cholecystectomy/gallbladder disease in relation to pancreatic cancer. Of the 11 studies

 *© 1999 Cancer Research Campaign*

indicating a positive association, only four demonstrated an increased pancreatic cancer risk 5 or more years prior to the cancer diagnosis (Norell and Ahlbom, 1986; Hyvarinen and Partanen, 1987; Shibata et al, 1994; Chow et al, 1998), while our study is the first to show an increased risk for subjects with a cholecystectomy at least 20 years prior to the cancer diagnosis, an interval too long to be considered prodromal to pancreatic cancer. A causal relation is further supported by experimental studies showing that cholecystectomy increases circulating levels of cholecystokinin (CCK), which is a major regulator of pancreatic growth and enzyme secretion (Warshaw and Fernandez-del Castillo, 1992), and a promoter of pancreatic carcinogenesis in rodents (Howatson and Carter, 1985; Smith et al, 1990). Potential confounding by obesity, which is a risk factor for cholelithiasis and pancreatic cancer, was ruled out in our study.

We observed little or no excess risk associated with duodenal or gastric ulcer or with gastrectomy as treatment for ulcer. Of 16 previous studies that have examined gastrectomy as a risk factor for pancreatic cancer, ten reported a positive association (McLean-Ross et al, 1982; Mack et al, 1986; Caygill et al, 1987; Offerhaus et al, 1987; Mills et al, 1988; Farrow and Davis, 1990; Tersmette et al, 1990; Eide et al, 1991; Mooller and Toftgaard, 1991; Bueno de Mesquita et al, 1992), while six studies in addition to ours found no association (Wynder et al, 1973; Maringhini et al, 1987; La Vecchia et al, 1990; Jain et al, 1991; Kalapothaki et al, 1993; Gullo et al, 1996). Of only four studies that examined history of ulcer in relation to pancreatic cancer risk, none found an association in accord with our findings (Mack et al, 1986; La Vecchia et al, 1990; Jain et al, 1991; Bueno de Mesquita et al, 1992).

A protective effect for allergic conditions including asthma has been reported in some studies of pancreatic cancer (Mack et al, 1986; Mills et al, 1988; Jain et al, 1991; Bueno de Mesquita et al, 1992; Kalapothaki et al, 1993; Dai et al, 1995), but our results are consistent with three studies that showed little effect (Gold et al, 1985; Farrow and Davis, 1990; La Vecchia et al, 1990). Since most case-control studies of pancreatic cancer have been based primarily on interviews with next of kin, the reliability of detailed information on allergic disorders may be questionable (Gavalda et al, 1995). Although we observed reduced risks for some conditions such as hay fever and allergies to animals and dust/moulds, no protective effect was seen for other types of allergies, so the findings may have been due to chance. In addition, the reduced risk for hay fever may have been due to the possible underestimation of the hay fever risk resulting from potential under-reporting of hay fever by interviewed cases as previously indicated in the Methods section.

The threefold risk associated with a family history of pancreatic cancer in first-degree relatives resembles the risk estimates reported in case-control studies in the USA (Falk et al, 1988), Canada (Ghadirian et al, 1991) and Italy (Fernandez et al, 1994). In addition, the risk of pancreatic cancer associated with familial occurrence was highest among long-term cigarette smokers, suggesting an interaction between smoking and genetic predisposition. Subjects with a family history of pancreatic cancer who smoked at least 20 years had a fivefold risk compared to a twofold risk among non-smokers and those who smoked for shorter durations. It is possible, however, that the differential familial risks are partly related to a tendency of smokers to have relatives who also smoke rather than to genetic predisposition. This possibility seems unlikely because there were no excesses associated with a family history of other smoking-related sites such as lung cancer. Alternatively, chance could not be reasonably excluded as an explanation for the differential familial risks by smoking status.

The elevated risk of pancreatic cancer extended to subjects with a family history of cancers of the colon (OR = 1.7), ovary (OR = 5.3), endometrium (OR = 1.5) and breast (OR = 1.3). These findings are consistent with the constellation of tumours associated with hereditary non-polyposis colon cancer (Lynch et al, 1985; Lumadue et al, 1995). This study is the first to report elements of the syndrome in a population-based study of pancreatic cancer, suggesting that the syndrome may occur more frequently than anticipated. In fact, 21% of cases compared to 13% of controls reported a first-degree relative with at least one of the types of cancer included in the syndrome, yielding a population attributable risk of 8% for the syndrome. Because tumour types were not confirmed in our study, these results need to be corroborated. The excess risk associated with a family history of liver cancer (OR = 1.4) is particularly questionable since it probably includes many more metastatic, rather than primary, liver cancers.

In summary, our population-based study indicates that diabetes and cholecystectomy are independent risk factors, as well as potential consequences of the malignancy, suggesting the role of metabolic factors in pancreatic cancer and opportunities for medical surveillance to detect early pancreatic cancer. We did not confirm the increased risk associated with gastrectomy or protective effects of allergies that have been reported in some, though not all, previous studies. The threefold risk of pancreatic cancer among first-degree relatives of affected individuals is consistent with previous studies, and the higher familial risk in smokers than non-smokers suggests the possibility of a gene–environment interaction. An increased risk of pancreatic cancer also was associated with a family history of colon, endometrium, ovary and breast cancer, suggesting a possible link to hereditary non-polyposis colon cancer.

### ACKNOWLEDGEMENTS

The authors thank Dr Jay Lubin for statistical advice; Ruth Thomson (Westat, Inc.) for her assistance in study management and coordination; Stella Semiti and Roy Van Dusen (Information Management Systems) for computer support; Judy Lichaa for clerical assistance; study coordinators, interviewers, and support staff in each study area for their diligent work; and the many physicians, hospitals, and study participants who cooperated in this study. This research was performed under National Cancer Institute contracts NO1-CP-51090, NO1-CP-51089, NO1-CP-51092, NO1-CN-05225, NO1-CN-31022 and NO1-CN-05227.

### REFERENCES

Anderson KE, Potter JD and Mack TM (1996) Pancreatic cancer. In: *Cancer Epidemiology and Prevention*, Schottenfeld D and Fraumeni JF Jr (eds), pp. 725–771. Oxford University Press: New York

Breslow NE and Day NE (1980) Vol I. Analysis of Case-Control Studies, Lyon. In: *Statistical Methods in Cancer Research*, pp. 5–338. IARC Scientific Publication: Lyon

Bueno de Mesquita HB, Maisonneuve P, Moerman CJ and Walker AM (1992) Aspects of medical history and exocrine carcinoma of the pancreas: a population-based case-control study in The Netherlands. *Int J Cancer* **52**: 17–23

Calle EE, Murphy TK, Rodriguez C, Thun MJ and Heath CW Jr (1998) Diabetes mellitus and pancreatic cancer mortality in a prospective cohort of United States adults. *Cancer Causes Control* **9**: 403–410

© 1999 Cancer Research Campaign 

Caygill CP, Hill MJ, Hall CN, Kirkham JS and Northfield TC (1987) Increased risk of cancer at multiple sites after gastric surgery for peptic ulcer. *Gut* **28**: 924–928

Chow W-H, Johansen C, Gridley G, Mellemkjaer L, Olsen JH and Fraumeni JF Jr (1998) Gallstones, cholecystectomy, and risk of cancers of the liver, biliary tract and pancreas. *Br J Cancer*

Chow W-H, Gridley G, Nyren O, Linet MS, Ekbom A, Fraumeni JF Jr and Adami HO (1995) Risk of pancreatic cancer following diabetes mellitus: a nationwide cohort study in Sweden. *J Natl Cancer Inst* **87**: 930–931

Colditz GA, Willett WC, Rotnitzky A and Manson JE (1995) Weight gain as a risk factor for clinical diabetes mellitus in women. *Ann Internal Med* **122**: 481–486

Cuzick J and Babiker AG (1989) Pancreatic cancer, alcohol, diabetes mellitus and gallbladder disease. *Int J Cancer* **43**: 415–421

Dai Q, Zheng W, Ji B-T, Shu X-O, Jin F, Zhu J-L and Gao Y-T (1995) Prior immunity-related medical conditions and pancreatic-cancer risk in Shanghai. *Int J Cancer* **63**: 337–340

DeFronzo RA, Bonadonna RC and Ferrannini E (1997) Pathogenesis of NIDDM. In: *International Textbook of Diabetes Mellitus*, Alberti KGMM, Zimmet P and DeFronzo RA (eds), pp. 635–701. John Wiley: New York

Dixon WJ, Brown MB, Engelmen L and Jennrick RI (eds) (1990) *BMDP Statistical Software Manual, Vol. II*. University of California Press: Berkeley, CA

Eide TJ, Viste A, Andersen A and Sooreide O (1991) The risk of cancer at all sites following gastric operation for benign disease. A cohort study of 4224 patients. *Int J Cancer* **48**: 333–339

Ekbom A, Yuen J, Karlsson BM, McLaughlin JK and Adami HO (1996) Risk of pancreatic and periampullar cancer following cholecystectomy. A population-based cohort study. *Dig Dis Sci* **41**: 387–391

Everhart J and Wright D (1995) Diabetes mellitus as a risk factor for pancreatic cancer. *JAMA* **273**: 1605–1609

Falk RT, Pickle LW, Fontham ET, Correa P and Fraumeni JF Jr (1988) Lifestyle factors for pancreatic cancer in Louisiana: a case-control study. *Am J Epidemiol* **128**: 324–336

Farrow DC and Davis S (1990) Risk of pancreatic cancer in relation to medical history and the use of tobacco, alcohol and coffee. *Int J Cancer* **45**: 816–820

Fernandez E, La Vecchia C, D’Avanzo B, Negri E and Franceschi S (1994) Family history and the risk of liver, gallbladder, and pancreatic cancer. *Cancer Epidemiol Biomark Prev* **3**: 209–212

Fisher WE, Boros LG and Schirmer WJ (1996) Insulin promotes pancreatic cancer: evidence for endocrine influence on exocrine pancreatic tumors. *J Surg Res* **63**: 310–313

Friedman G and van den Eeden SK (1993) Risk factors for pancreatic cancer: an exploratory study. *Int J Epidemiol* **22**: 30–37

Gavalda L, Porta M, Malats N, Pinol JL, Fernandez E, Maguire A, Cortes I, Carrillo E, Marrugat M, Rifa J and Carrato A (1995) Concordancia entre la información facilitada por el paciente y un familiar sobre antecedentes patológicos, consumo de tabaco, de alcohol, de café, y dieta en el cancer de páncreas exocrino y del sistem biliar extrahepático. *Gac Sanit* **9**: 334–342

Ghadirian P, Boyle P, Simard A, Baillargeon J, Maisonneuve P and Perret C (1991) Reported family aggregation of pancreatic cancer within a population-based case-control study in the Francophone community in Montreal, Canada. *Int J Pancreatol* **10**: 183–196

Gold EB, Gordis L, Diener MD, Seltser R, Boitnott JK, Bynum TE and Hutcheon DF (1985) Diet and other risk factors for cancer of the pancreas. *Cancer* **55**: 460–467

Green A and Jensen OM (1985) Frequency of cancer among insulin-treated diabetic patients in Denmark. *Diabetologia* **28**: 128–130

Gullo L, Pezzilli R and Morselli-Labate AM (1996) Italian Pancreatic Cancer Study Group. Risk of pancreatic cancer associated with cholelithiasis, cholecystectomy, or gastrectomy. *Dig Dis Sci* **41**: 1065–1068

Howatson AG and Carter DC (1985) Pancreatic carcinogenesis: enhancement of cholecystokinin in the hamster-nitrosamine model. *Br J Cancer* **51**: 107–114

Hyvarinen H and Partanen S (1987) Association of cholecystectomy with abdominal cancers. *Hepato-gastroenterology* **34**: 280–284

Jain M, Howe GR, St Louis P and Miller AB (1991) Coffee and alcohol as determinants of risk of pancreas cancer: a case-control study from Toronto. *Int J Cancer* **47**: 384–389

Ji B-T, Hatch MC, Chow W-H, McLaughlin JK, Dai Q, Howe GR, Gao Y-T and Fraumeni JF Jr (1996) Anthropometric and reproductive factors and the risk of pancreatic cancer: a case-control study in Shanghai, China. *Int J Cancer* **66**: 432–437

Johansen C, Chow W-H, Jorgensen T, Mellemkjaer L, Engholm G and Olsen JH (1996) Risk of colorectal cancer and other cancers in patients with gall stones. *Gut* **39**: 439–443

Kalapothaki V, Tzonou A, Hsieh C, Toupadaki N, Karakatsani A and Trichopoulos D (1993) Tobacco, ethanol, coffee, pancreatitis, diabetes mellitus, and cholelithiasis as risk factors for pancreatic carcinoma. *Cancer Causes Control* **4**: 375–382

Kazakoff K, Cardesa T, Liu J, Adrian TE, Bagchi D, Bagchi M, Birt DF and Pour PM (1996) Effects of voluntary physical exercise on high-fat diet-promoted pancreatic carcinogenesis in the hamster model. *Nutr Cancer* **26**: 265–279

La Vecchia C, Negri E, D’Avanzo BD, Ferraroni M, Gramenzi A, Savoldelli R, Boyle P and Franceschi S (1990) Medical history, diet and pancreatic cancer. *Oncology* **47**: 463–466

Landis SH, Murray S and Wingo PA (1998) Cancer statistics, 1998. *Cancer J Clin* **48**: 6–29

Lin RS and Kessler II (1981) A multifactorial model for pancreatic cancer in man. *JAMA* **245**(2): 147–152

Lumadue JA, Griffin CA, Osman M and Hruban RH (1995) Familial pancreatic cancer and the genetics of pancreatic cancer. *Surg Clin N Am* **75**(5): 845–855

Lynch HT, Voorhees GJ, Lanspa SJ, McGreevy PS and Lynch JF (1985) Pancreatic carcinoma and hereditary non-polyposis colorectal cancer: a family study. *Br J Cancer* **52**: 271–273

Mack TM, Yu MC, Hanisch R and Henderson BE (1986) Pancreas cancer and smoking, beverage consumption, and past medical history. *J Natl Cancer Inst* **76**: 49–60

Maringhini A, Thiruvengadam R, Melton LJ, Hench VS, Zinsmeister AR and DiMagno EP (1987) Pancreatic cancer risk following gastric surgery. *Cancer* **60**: 245–247

McLaughlin JK, Mandel JS, Mehl ES and Blot WJ (1990) Comparison of next-of-kin with self-respondents regarding questions on cigarette, coffee and alcohol consumption. *Epidemiology* **1**: 408–412

McLean-Ross AH, Smith MA, Anderson JR and Small WP (1982) Late mortality after surgery for peptic ulcer. *N Engl J Med* **307**: 519–522

Micozzi MS, Albanes D, Jones DY and Chumlea WC (1986) Correlations of body mass indices with weight, stature, and body composition in men and women in NHANES I and II. *Am J Clin Nutr* **44**: 725–731

Mills, PK, Beeson WL, Abbey DE, Fraser GE and Phillips RL (1988) Dietary habits and past medical history as related to fatal pancreas cancer risk among Adventists. *Cancer* **61**: 2578–2585

Moller H, Mellemgaard A, Lindvig K and Olsen JH (1994) Obesity and cancer risk: a Danish record-linkage study. *Eur J Cancer* **30a**: 344–350

Mooller H and Toftgaard C (1991) Cancer occurrence in a cohort of patients surgically treated for peptic ulcer. *Gut* **32**: 740–744

Norell S and Ahlbom A (1986) Diabetes, gall stone disease, and pancreatic cancer. *Br J Cancer* **54**: 377–378

Offerhaus JGA, Giardiello FM, Moore GW and Tersmette AC (1987) Partial gastrectomy: a risk factor for carcinoma of the pancreas? *Hum Pathol* **18**(3): 285

Permert J, Larsson J, Westermark GT, Herrington MK, Christmanson L, Pour PM, Westermark P and Adrian TE (1994) Islet amyloid polypeptide in patients with pancreatic cancer and diabetes. *N Engl J Med* **330**: 313–318

Pour PM and Kazakoff K (1996) Stimulation of islet cell proliferation enhances pancreatic ductal carcinogenesis in the hamster model. *Am J Pathol* **149**: 1017–1025

Shibata A, Mack TM, Paganini-Hill A, Ross RK and Henderson BE (1994) A prospective study of pancreatic cancer in the elderly. *Int J Cancer* **58**: 46–49

Silverman DT, Brown LM, Hoover RN, Schiffman M, Lillemoe KD, Schoenberg JB, Swanson GM, Hayes RB, Greenberg RS, Benichou J and Schwartz AG (1995) Alcohol and pancreatic cancer in blacks and whites in the United States. *Cancer Res* **55**: 4899–4905

Silverman DT, Dunn J, Hoover R, Schiffman M, Lillemoe KD, Schoenberg JB, Brown LM, Greenberg RS, Hayes RB, Swanson GM, Wacholder S, Schwartz AG, Liff JM and Pottern LM (1994) Cigarette smoking and pancreas cancer: a case-control study based on direct interviews. *J Natl Cancer Inst* **86**: 1510–1516

Silverman DT, Swanson CA, Gridley G, Wacholder S, Greenberg RS, Brown LM, Hayes RB, Swanson GM, Schoenberg JB, Pottern LM, Schwartz AG, Fraumeni JF Jr and Hoover RN (1998) Dietary and nutritional factors and pancreatic cancer: a case-control study based on direct interviews. *J Natl Cancer Inst* **90**: 1710–1719

Silverman DT, Schiffman M and Devesa SS (1996) Diagnostic certainty in pancreatic cancer. *J Clin Epidemiol* **49**: 601–603

Smith JP, Solomon TE, Bagheri S and Kramer S (1990) Cholecystokinin stimulates growth of human pancreatic adenocarcinoma SW-1990. *Dig Dis Sci* **35**: 1377–1384

 *© 1999 Cancer Research Campaign*

Tersmette A, Offerhaus JA, Giardiello FM and Vandenbroucke JP (1990) Occurrence of non-gastric cancer in the digestive tract after remote partial gastrectomy: analysis of an Amsterdam cohort. *Int J Cancer* **46**: 792–795

Waksberg J (1978) Sampling methods for random digit dialing. *J Am Stat Assoc* **73**: 40–46

Warshaw AL and Fernandez-del Castillo C (1992) Pancreatic carcinoma. *N Engl J Med* **326**: 455–465

Wideroff L, Gridley G, Mellemkjaer L, Chow WH, Linet M, Keehn S, Borch-Johnsen K and Olsen JH (1997) Cancer incidence in a population-based cohort of patients hospitalized with diabetes mellitus in Denmark. *J Natl Cancer Inst* **89**: 1360–1365

Wynder EL, Mabuchi K, Maruchi N and Fortner JG (1973) Epidemiology of cancer of the pancreas. *J Natl Cancer Inst* **50**: 645–667

*© 1999 Cancer Research Campaign*
